# Review: Contribution of transgenic models to understanding human prion disease

**DOI:** 10.1111/j.1365-2990.2010.01129.x

**Published:** 2010-12

**Authors:** J D F Wadsworth, E A Asante, J Collinge

**Affiliations:** MRC Prion Unit and Department of Neurodegenerative Disease, Institute of Neurology, University College London, National Hospital for Neurology and NeurosurgeryLondon, UK

**Keywords:** Creutzfeldt-Jakob disease, fatal familial insomnia, Gerstmann-Sträussler-Scheinker disease, kuru, prion, variant Creutzfeldt-Jakob disease

## Abstract

J. D. F. Wadsworth, E. A. Asante and J. Collinge (2010) *Neuropathology and Applied Neurobiology***36,** 576–597**Contribution of transgenic models to understanding human prion disease**

Transgenic mice expressing human prion protein in the absence of endogenous mouse prion protein faithfully replicate human prions. These models reproduce all of the key features of human disease, including long clinically silent incubation periods prior to fatal neurodegeneration with neuropathological phenotypes that mirror human prion strain diversity. Critical contributions to our understanding of human prion disease pathogenesis and aetiology have only been possible through the use of transgenic mice. These models have provided the basis for the conformational selection model of prion transmission barriers and have causally linked bovine spongiform encephalopathy with variant Creutzfeldt-Jakob disease. In the future these models will be essential for evaluating newly identified potentially zoonotic prion strains, for validating effective methods of prion decontamination and for developing effective therapeutic treatments for human prion disease.

## Introduction

Prions have attracted immense research interest for many years because of their unique composition and properties – being apparently devoid of significant nucleic acid [1–5]. According to the widely accepted ‘protein-only’ hypothesis [6], host-encoded cellular prion protein (PrP^C^) is converted to an alternative form designated PrP^Sc^[1–5]. It is proposed that PrP^Sc^ is the infectious agent acting to replicate itself with high fidelity by recruiting endogenous PrP^C^ and that the difference between these isoforms lies purely in the monomer conformation and its state of aggregation [1–5,7–9]. Notably however, although abnormal isoforms of PrP are undoubtedly the major constituent of mammalian prions, it has not yet been excluded that other molecules may contribute to infectious prion composition or may be required to direct the assembly of PrP^Sc^[10–12]. In this regard the precise identification of minor ‘contaminants’ that co-purify with PrP^Sc^ may still be of critical importance to understanding infectious prion composition, the determinants of prion strain or the ability of a prion to infect a host [13].

Central to understanding prion propagation remains the conundrum of prion strains – how a protein-only infectious agent can encode information required to specify distinct disease phenotypes – and also the so-called species barrier effect which limits cross species infection. While originally considered different aspects of the prion problem it is now clear that species barriers and prion strains are intimately related by ‘conformational selection’[5,14]. This hypothesis proposes that although a wide range of mammalian PrP^Sc^ conformations may be possible, only a subset will be compatible with each individual PrP primary structure. Ease of transmission of prions between species (or also within species as a result of PrP polymorphisms), therefore relates to overlap of permissible PrP^Sc^ conformations between the structures of PrP from the source and recipient as well as heterogeneity in cellular mechanisms affecting prion propagation and clearance kinetics [5,14]. Importantly, conformational selection has now been strongly supported by elegant studies of prions in yeast and other fungi [15–17] and intriguingly evidence for strains in Alzheimer's disease is also now emerging, with self-propagating variations in the structure of amyloid-β fibrils appearing to correlate with differences in cyto-toxicity [18] and patterns of amyloid deposition in transgenic mice [19]. Elucidation of the composition and structure of infectious mammalian prions will therefore not only provide a major advance to understanding the molecular mechanism of prion replication, with direct translational benefits for both diagnosis and rational therapeutics, but will also be of great relevance to a wide range of other neurodegenerative diseases involving accumulation of misfolded host proteins [5,20,21]. Indeed, evidence for commonality of structural features in protein misfolding diseases is provided already by antibodies raised against oligomeric forms of PrP which detect soluble oligomeric forms of a number of other amyloid proteins [22]. More recently it has been proposed that PrP^C^ may play a critical role in the pathogenesis of Alzheimer's disease by mediating amyloid-β oligomer induced synaptic dysfunction [23].

Aside from the intrinsic biological interest of studying prions, human prion disease is a strategic priority for public health protection. The occurrence of variant Creutzfeldt-Jakob disease (vCJD) [24] and the experimental confirmation that it is caused by the same prion strain as that causing bovine spongiform encephalopathy (BSE) in cattle [25–28], has dramatically established the zoonotic potential of animal prion diseases. The extremely prolonged and variable incubation periods seen in human prion disease and the possibility of subclinical carrier states means that it will be some years before the full consequences of human exposure to BSE prions are known [14,29–33]. In the meantime, we are faced with the possibility that significant numbers in the population may be incubating this disease and that they might pass it on to others via blood transfusion, blood products, tissue and organ transplantation and other iatrogenic routes [14,33–40]. Notably, while cattle BSE is now effectively controlled, the emergence of other new or newly recognized potentially zoonotic animal prion strains remains a key issue for public health. A number of novel isolates of bovine prion disease have now been identified which appear to be distinct prion strains [41–44] and the host range of atypical sheep prions [43,45,46] has not been established. Because prion strains can adapt and mutate on passage in new species [5,43,47], and also within species as a result of PrP polymorphisms and other genetic factors [28,48–51], the evaluation of their risks to public health is complex. The demonstration of subclinical carrier states of prion infection in animal models is also relevant to public health, both with respect to prion zoonoses and iatrogenic transmission of human prions [28,33,39,52]. Prions resist many conventional sterilization procedures and effective methods for prion decontamination of surgical instruments and medical equipment although reported have yet to be effectively implemented [53,54].

In order to understand the molecular basis of human prion disease, develop rational therapeutics, improved decontamination methods and diagnostic tools, effective and appropriate experimental models are essential. However, very few alternative experimental approaches are available for studying prion diseases as incubation time, clinical phenotype, neuropathology, immune responses and behaviour can only be studied in an animal. Early studies of human prions used primates [55–57]; however, following the demonstration in 1995 that the species barrier limiting transmission of human prions to wild-type mice can be obviated by expression of human PrP in the absence of endogenous mouse PrP [58,59] such ‘humanised’ transgenic mice have become key experimental models for studying human prion disease [43,60–65]. Two types of genetic modification can be used to generate human PrP-expressing mice, either transgenic expression of human PrP on a mouse PrP knockout background [62] or direct replacement of mouse PrP with human PrP using gene knock-in technology [61].

## Determinants of phenotypic variability in human prion disease

**Figure 1:**
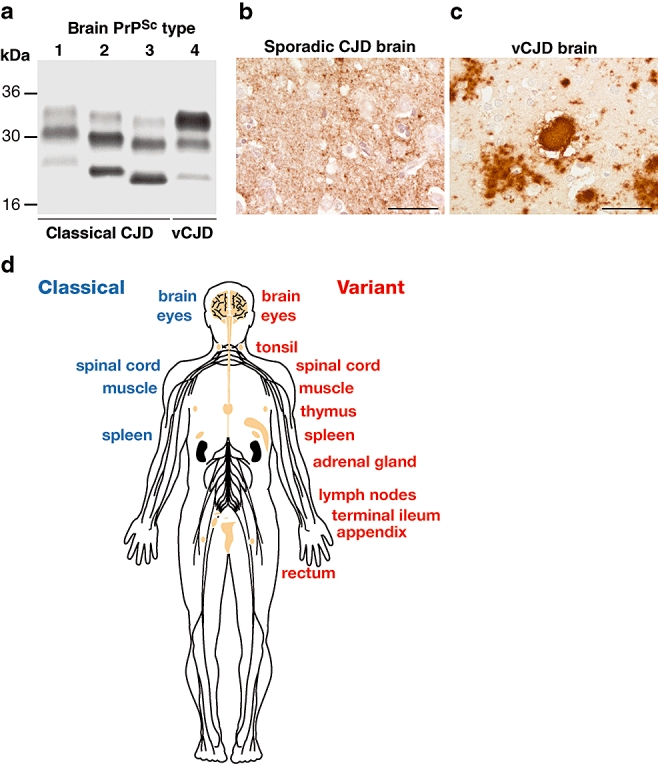
Variant Creutzfeldt-Jakob disease (vCJD) is a distinct human prion strain. (**a**) Immunoblot of proteinase K digested brain homogenates using antiPrP monoclonal antibody 3F4 showing PrP^Sc^ types 1–4 in human brain according to the London classification [90]. Types 1–3 PrP^Sc^ are seen in the brain of classical forms of CJD (either sporadic or iatrogenic CJD) and kuru, while type 4 PrP^Sc^ is uniquely seen in vCJD brain [25,90,122]. (**b,c**) Brain sections from sporadic CJD (**b**) and vCJD (**c**) showing abnormal PrP accumulation following immunohistochemistry using antiPrP monoclonal antibody ICSM35. Abnormal PrP deposition in sporadic CJD most commonly presents as diffuse, synaptic staining, whereas vCJD is distinguished by the presence of florid PrP plaques consisting of a round amyloid core surrounded by a ring of spongiform vacuoles. Scale bars: 50 µm. (**d**) Distribution of PrP^Sc^ in human tissues. The schematic diagram shows tissues in which PrP^Sc^ has been detected using high sensitivity immunoblotting. The vCJD has a peripheral pathogenesis distinct from classical forms of CJD, with a prominent and uniform involvement of lymphoreticular tissues.

Human prion diseases include Creutzfeldt-Jakob disease (CJD), Gerstmann-Sträussler-Scheinker disease (GSS), fatal familial insomnia, kuru and vCJD in humans [1,2,39]. They are associated with a range of clinical presentations and are classified by both clinico-pathological syndrome and aetiology with subclassification according to molecular criteria [39,66] (Table 1). The clinical presentation of human prion disease varies enormously and there is considerable overlap observed between individuals with different disease aetiologies [33,39,66,67] and even in family members with the same pathogenic *PRNP* mutation [67–74]. Remarkably, kuru demonstrates that incubation periods of infection with human prions can exceed 50 years [32,75]. Progressive dementia, cerebellar ataxia, pyramidal signs, chorea, myoclonus, extrapyramidal features, pseudobulbar signs, seizures and amyotrophic features can be seen in variable combinations. Criteria used for diagnosis of human prion disease have been defined [39,76] and definite diagnosis of sporadic and acquired prion disease relies upon neuropathological examination and the demonstration of pathological PrP deposition in the central nervous system by either immunoblotting or immunohistochemistry [39,76–79] (Figure 1). Polymorphism at residue 129 of human PrP [encoding either methionine (M) or valine (V)] powerfully affects susceptibility to human prion diseases [80–85]. About 38% of northern Europeans are homozygous for the more frequent methionine allele, 51% are heterozygous, and 11% homozygous for valine. Homozygosity at *PRNP* codon 129 predisposes to the development of sporadic and acquired CJD [80–85] and is most strikingly observed in vCJD where all neuropathologically confirmed cases studied so far have been homozygous for codon 129 methionine of *PRNP*[38,39,49,50].

**Table 1:** Classification of human prion disease

*Aetiology*	*Phenotype*	*Frequency*	*References*
Sporadic			
Unknown: random distribution worldwide; incidence of 1–2 per million per annum	Sporadic CJD: subacute myoclonic form and range of atypical forms; multiple distinct prion strains associated with distinct clinicopathological phenotypes which include sporadic fatal insomnia	Approximately 85%	[39,66,85,217]
Inherited			
Autosomal dominantly inherited conditions with high penetrance; all forms have germline *PRNP* coding mutations	Extremely variable: readily mimics familial Alzheimer's disease and other neurodegenerative conditions; over 30 mutations identified; includes GSS, familial CJD and FFI	10–15%	[39,66,67,72]
Acquired			
Iatrogenic infection with human prions via medical or surgical procedures; cadaveric derived pituitary hormones, tissue grafts, and contaminated neurosurgical instruments	Iatrogenic CJD: typical CJD when direct central nervous system human exposure; ataxic onset when peripheral infection	<5% (most from USA, UK, France and Japan)	[39,120,225]
Exposure to human prions via endocannibalism	Kuru	Unique to small area of Papua New Guinea; major epidemic in 1950s with gradual decline since cessation of cannibalism	[32,39,75,226,227]
Exposure (presumed dietary) to BSE prion strain; probable secondary transmission via blood transfusion and possibly blood products	Variant CJD	Mainly UK with patients in several other countries	[14,24,33,39,40]

BSE, bovine spongiform encephalopathy; CJD, Creutzfeldt-Jakob disease; FFI, fatal familial insomnia; GSS, Gerstmann-Sträussler-Scheinker disease.

Prion strains are classically distinguished by distinct incubation periods and by patterns of neuropathological targeting (so-called lesion profiles) in a panel of defined inbred mouse lines [86]. Common histopathological features involve a classical triad of spongiform vacuolation (affecting any part of the cerebral grey matter), neuronal loss, and astrocytic and microglial proliferation and may be accompanied by amyloid plaques composed of insoluble aggregates of PrP [77,87]. Amyloid plaques are a notable feature of kuru and GSS [77,88,89] but they are less frequently found in the brains of patients with sporadic CJD which typically show a diffuse pattern of PrP deposition [77,90] (Figure 1). The histopathological features of vCJD are relatively consistent when compared to sporadic CJD and distinguish it from other human prion diseases. The most distinctive feature is the presence of large numbers of PrP-positive amyloid plaques that differ in morphology from the plaques seen in kuru and GSS in that the surrounding tissue takes on a microvacuolated appearance, giving the plaques a florid appearance [24,91] (Figures 1 and 2).

## Difficulties in assigning human prion strains

The hypothesis that alternative conformations or assembly states of PrP^Sc^ provide the molecular substrate for a significant part of the clinicopathological heterogeneity seen in human prion diseases and that this relates to the existence of distinct human prion strains is supported by considerable experimental evidence [1–5,25,92–95] and also by the demonstration of protein conformation-based inheritance mechanisms of yeast prions [15–17]. Despite these advances, the precise molecular basis of mammalian prion strain diversity is unknown. A major confounding issue in this regard has been in resolving whether relatively subtle biochemical differences in PrP^Sc^ are of biological importance and accurately reflect the propagation of distinct human prion strains. This is particularly true in sporadic CJD [25,90,93,96–100] where progress has been severely hampered by a lack of transgenic modelling data to firmly distinguish the identity of distinct prion strains and their defining molecular and neuropathological phenotypes. This fundamental problem coupled with the difficulties and variability of the biochemical methods used to distinguish PrP^Sc^ types [90,96–98,100–102] has so far precluded an internationally accepted classification system for human prion strains. In this regard, the increasingly recognized co-occurrence of different PrP^Sc^ types in the same brain [74,85,90,93,102–108] and the recognition that protease-sensitive pathological isoforms of PrP (distinct from prototypical PrP^Sc^) may have a significant role in both animal and human prion disease [94,99,109–116] has further confounded progress. All of these factors, together with the known ability of genetic background to influence prion strain selection [28,50,51,117–119] and knowledge that route of transmission in acquired human prion disease may dramatically influence clinical and neuropathological presentation [120–123], has re-emphasized the requirement to remove host variability by identifying distinct prion strains in appropriate transgenic models.

## Transgenic modelling has made key contributions to understanding prion biology

Considerable evidence argues that prions are composed largely, if not entirely, of abnormal isoforms of PrP [1–3,5,8]. The essential role of host PrP for prion propagation and pathogenesis is demonstrated by the fact that knockout mice lacking the PrP gene (*Prnp°^/^°* mice) are entirely resistant to prion infection [124,125] and that reintroduction of PrP transgenes restores susceptibility to infection in a species-specific manner that allows reverse genetics approaches to studying structure-function relationships in PrP (for reviews see [62,65,126]). PrP in its entirety is unnecessary for prion propagation. Not only can the unstructured N-terminal 90 amino acids be deleted [127,128], but also the first α-helix, the second β-strand and part of helix 2. In transgenic animals, a 106 amino acid fragment of the protein comprising PrPΔ23–88 and Δ141–176 conferred susceptibility to and propagation of prions [129,130]. Notably while expression of PrP N-terminal deletion mutants to residue 106 are tolerated and support prion propagation [127,128], deletion beyond this leads to severe ataxia and neuronal loss in the granular cell layer of the cerebellum [62,131]. Intriguingly, the Doppel protein (Dpl) [132], which has a similar structure to N-terminally truncated PrP, causes a similar cerebellar effect when ectopically expressed in the brain [133]. The severity of neurotoxicity correlates with the level of Dpl expression [134] and can be rescued by PrP^C^ expression [135], indicating that PrP^C^, Dpl and ΔPrP might compete for a common hypothetical receptor or ligand L_PrP_ that transduces neuroprotective signals when bound to PrP^C^ but not when bound to Dpl or ΔPrP [62,131,136,137]. This model also proposes the existence of a PrP^C^-like protein termed Π that is capable of compensating for the absence of PrP^C^ in *Prnp*°^/^° mice. Recently the protein Sho has been demonstrated to be a glycosylphosphatidylinositol (GPI)-anchored neuronal glycoprotein present in the central nervous system (CNS) from early postnatal life that can counteract the neurotoxic effects of either Dpl or ΔPrP and is therefore a candidate for Π[138].

While PrP expression is absolutely required for prion propagation and neurotoxicity [124] knockout of PrP^C^ in embryonic models [139,140] or in adult brain [141] has no overt phenotypic effect that influences lifespan or fertility. These findings demonstrate that acute loss of PrP^C^ in neurones in adulthood is tolerated, and that the neuropathophysiology of prion diseases is not due to loss of PrP^C^ function [142,143]. *Prnp°^/^°* mice are not normal, however (for reviews see [2,62,144,145]). In particular, in addition to a role for PrP^C^ in providing neuroprotective signals, abnormalities in synaptic physiology, circadian rhythms, cognition and olfactory physiology have been reported [146–153]. Notably, a reduction of slow afterhyperpolarizations evoked by trains of action potentials in hippocampal neurones in *Prnp°^/^°* mice due to disruption of calcium-activated potassium currents is also affected by the conditional knockout of PrP^C^ suggesting that this phenotype is specifically due to the absence of PrP^C^, reflecting loss of a differentiated neuronal function, rather than a developmental deficit arising from congenital knockout of PrP^C^[141]. Important functional correlates of abnormalities of synaptic transmission in *Prnp°^/^°* mice include cognitive deficits [152] and impairment of olfactory physiology and behaviour [153] which can be rescued by transgenic neuronal expression of PrP^C^. Very recently it has been revealed that axonal PrP^C^ expression is required for peripheral myelin maintenance [154] and this finding correlates strongly with earlier demonstrations of extensive demyelination in transgenic mice expressing PrP with deletion mutants in the central domain [155–157]. Importantly, despite current uncertainties regarding the conversion of PrP^C^ to PrP^Sc^ and possible mechanisms of neurotoxicity [5], the prevention of this conversion in neurones by conditional knock out of PrP^C^ has been shown to prevent disease progression and reverse early degenerative changes [142]. These data have firmly established PrP^C^ as the prime target for rational therapeutics in prion disease [143,158,159]. Conversely, although PrP^Sc^ has long been considered as a target for chemotherapy [158], drugs interacting with PrP^Sc^ are likely to be prion strain-specific and may only target a specific subset of PrP^Sc^ conformers resulting in propagation of drug resistant prions [143,160].

## Human PrP transgenic mouse models

**Figure 3:**
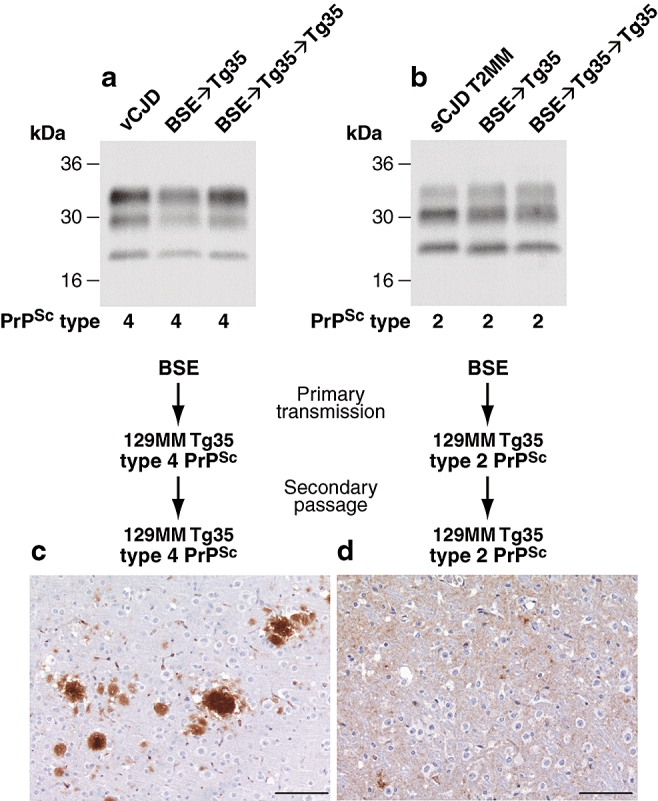
Bovine spongiform encephalopathy (BSE) prions propagate as either variant Creutzfeldt-Jakob disease (vCJD)-like or sporadic CJD (sCJD)-like strains in transgenic mice expressing human prion protein. Primary transmission of BSE prions in transgenic Tg(HuPrP129M^+/+^*Prnp°^/^°*)-35 mice (Tg35) results in the propagation of either type 4 PrP^Sc^ and the occurrence of abundant florid PrP plaques that are the neuropathological hallmark of vCJD or type 2 PrP^Sc^ and the occurrence of diffuse PrP deposition that is typically seen in sporadic CJD [28]. Molecular and neuropathological characteristics of these distinct prion strains remain stable after secondary passage in the same line of transgenic mice [49]. (**a,b**) Representative immuno-blots of proteinase-K treated brain homogenates from vCJD and sCJD (*PRNP* 129 MM genotype with type 2 PrP^Sc^; sCJD T2MM) and transgenic mice analysed with antiPrP monoclonal antibody 3F4. The identity of the brain sample is designated above each lane with the type of PrP^Sc^ present in the sample designated below, using the London classification [90]. (**c**) Representative immunohistochemical analysis of transgenic mouse brain (thalamus) at secondary passage showing abnormal PrP immunoreactivity, including PrP-positive plaques, stained with antiPrP monoclonal antibody ICSM 35. Scale bars: 100 µm.

Sporadic CJD prions transmit disease only occasionally to wild-type mice with long and variable incubation periods [26,58,59,122,161]. Early attempts to transmit human prions to transgenic mice met with varied success. Tg(HuPrP)110 and Tg(HuPrP)152 transgenic lines were made by co-expressing wild-type human PrP with valine at codon 129 in mice also expressing endogenous mouse PrP^C^[161]. However after inoculation with brain homogenates from patients with GSS and sporadic and iatrogenic CJD these transgenic recipients showed no higher frequency of disease than inoculated nontransgenic control mice. This lack of susceptibility of Tg(HuPrP) mice, together with the earlier pioneering work of Scott *et al*. [162], subsequently led Telling and colleagues to generate mice that expressed a chimeric PrP protein in which a segment of mouse PrP^C^ was replaced with the corresponding human PrP sequence [161]. When similarly inoculated with CJD prions, all the Tg(MHu2MPrP) mice developed neurological disease around 200 days post-inoculation [161]. Concomitant with this chimeric transgene approach, the endogenous mouse PrP allele was also removed by breeding Tg(HuPrP)152 mice to PrP null mice (*Prnp°^/^°*) to produce homozygous Tg(HuPrP)152/Prnp°^/^°[58,59]. These mice were found to be highly susceptible to CJD prions, with all inoculated mice succumbing to disease at short incubation periods [58,59]. Although the chimeric transgene approach has provided extremely important advances to our understanding of human prion strain propagation [59,92,161,163,164], the demonstration that the human to mouse transmission barrier is overcome simply by expressing human PrP^C^ in the absence of endogenous mouse PrP^C^[58,59] has established this as the most straightforward approach for investigating human prion strain diversity. Importantly, this approach enables the effects of genotype in the inoculum and recipient transgenic mouse to be modelled definitively to provide key information on the role of human PrP primary structure in influencing prion strain propagation [2,5,33,60]. More recently, other human PrP^C^-expressing mouse models have been generated using knock-in technology which allows transgene integration at the normal genomic location and endogenous levels of expression under the control of normal gene regulatory elements [61,63,165–167]. Both transgenic and knock-in modelling of prion diseases provide complimentary results in most cases; however, over-expression of human PrP transgenes may be desirable. Over-expression results in considerable truncation in incubation periods such that these models are more convenient in practice and in some instances transmission may require over-expression. For instance, transmission of BSE prions to human PrP knock-in mice resulted in no infection [63] whereas the transgenic approach has clearly demonstrated that BSE infection results in complete recapitulation of the vCJD phenotype [28] (Figure 3). Further, it is known that human PrP^C^ functions less efficiently in mice than mouse PrP^C^, as over-expression is required to rescue a PrP null phenotype [168] and so ‘endogenous’ levels of heterologous PrP^C^ expression may not in fact be the best model of susceptibility.

## Transgenic modelling of sporadic and acquired human prion disease

Humanized transgenic mice expressing human PrP 129 valine on a *Prnp* null background are highly susceptible to sporadic CJD prions regardless of the PrP^Sc^ type or codon 129 genotype of the inoculum [25,26,58,59,122,164,169]. These transmissions are typically characterized by 100% attack rates of prion infection producing uniform clinical prion disease after similar short incubation periods of around 200 days [25,26,58,59,122,164,169]. In isolates that have been examined, no fall in mean incubation period is seen after secondary passage in the same mice indicative of the lack of a transmission barrier [58]. The absence of a transmission barrier to sporadic CJD prions is not, however, uniformly observed in transgenic mice expressing human PrP 129 methionine on a *Prnp* null background. Here mismatch at residue 129 between the inoculum and host can significantly affect transmission. Thus while there appears to be no barrier to transmission of sporadic CJD prions from codon 129 methionine homozygous patients [28,41,164,170], transmission of sporadic CJD prions from codon 129 heterozygous patients and 129 valine homozygous patients is often associated with more prolonged and variable incubation periods and reduced attack rates [28,164,169]. Consistent with both aetiology and the occurrence of the same PrP^Sc^ types that are seen in the brain of sporadic CJD patients, iatrogenic CJD prions [25,26,164,169,171] and kuru prions [122] have transmission properties equivalent to those of sporadic CJD prions. Although the precise number of distinct prion strains that are propagated in sporadic CJD remains unknown, Manson and colleagues have recently presented evidence for four distinct prion strains from a limited number of sporadic CJD patients using human PrP knock-in mice [167].

**Figure 2:**
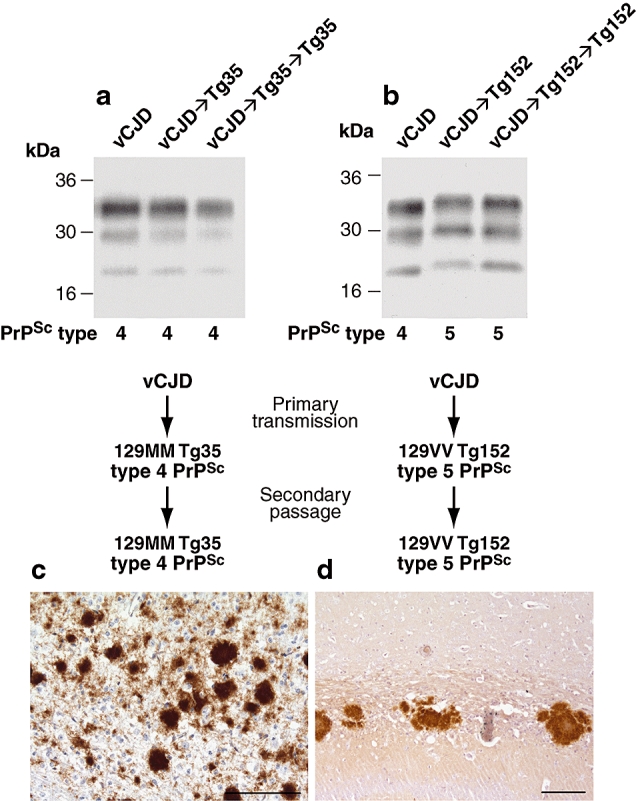
Human prion protein with valine at residue 129 prevents expression of the variant Creutzfeldt-Jakob disease (vCJD) phenotype. Primary and secondary transmission of vCJD prions to transgenic Tg(HuPrP129M^+/+^*Prnp°^/^°*)-35 mice (Tg35) results in faithful propagation of type 4 PrP^Sc^ and the occurrence of abundant florid PrP plaques throughout the cortex that are the neuropathological hallmark of vCJD [49]. In contrast, primary transmission of vCJD prions to transgenic Tg(HuPrP129V^+/+^*Prnp°^/^°*)-152 mice (Tg152) produces a novel prion strain that is maintained on secondary passage in the same mice distinguished by the propagation of type 5 PrP^Sc^ and a distinct pattern of neuropathology characterized by large nonflorid PrP plaques restricted to the corpus callosum [26,49]. (**a,b**) Representative immuno-blots of proteinase-K treated brain homogenates from variant CJD and transgenic mice analysed with antiPrP monoclonal antibody 3F4. The identity of the brain sample is designated above each lane with the type of PrP^Sc^ present in the sample designated below using the London classification [90]. (**c,d**) Representative immunohistochemical analysis of transgenic mouse brain at secondary passage showing abnormal PrP plaques stained with antiPrP monoclonal antibodies ICSM 35 (**a**) or 3F4 (**b**). Scale bars: 100 µm.

In contrast, to prions propagated in classical CJD and kuru the transmission properties of vCJD prions are strikingly distinct and have established vCJD as a distinct human prion strain (Figures 1 and 2). Our research was the first to demonstrate transmission of BSE prions to transgenic mice expressing human PrP and these data confirmed that vCJD was caused by human exposure to the BSE prion strain [26,28] (Figure 3). The vCJD prions transmit disease to wild-type mice far more efficiently than any other form of human prion disease [26,27,49,122] and in transgenic mice faithful propagation of the vCJD phenotype is dependent upon homozygous expression of human PrP 129 methionine [28,49,63,170,171] (Figure 2). Transgenic mice homozygous for human PrP 129 valine show a pronounced transmission barrier to vCJD prions [26,49,63,166] and propagate a distinct prion strain that has not yet been observed in humans [26,49,171] (Figure 2). Because human PrP with 129 valine appears to be incompatible with the PrP^Sc^ conformation propagated in vCJD [49] (Figure 2), and may have a dominant negative influence on the propagation of the vCJD prion strain in codon 129 heterozygous mice [171,172], this could explain why all neuropathologically confirmed cases of vCJD have been in individuals homozygous for 129 methionine. While caution must be exercised in extrapolating from animal models, even where faithful recapitulation of molecular and pathological phenotypes is possible [28,49,122,171], our findings, together with more recent studies from other laboratories [63,170], argue that primary human BSE prion infection, and secondary infection through iatrogenic routes, will not be restricted to a single disease phenotype. Dependent upon the genotype of the prion source and the recipient, the propagation of prion strains seen in sporadic CJD or other novel prion strain types are anticipated [28,33,49,122,171] (Figures 2 and 3). These data reiterate the need to continue to stratify all human prion disease patients at the molecular level to facilitate rapid recognition of novel subtypes and change in the relative frequencies of particular subtypes due to either BSE exposure patterns or iatrogenic sources of human prions.

## Conformational selection dictates human prion strain propagation

Homozygosity at polymorphic residue 129 of human PrP^C^ remains the key genetic susceptibility factor for sporadic and acquired prion disease [50,80–84] and in vCJD it represents the strongest known common genetic susceptibility polymorphism in any human disease [39,50,173]. The transgenic studies described above have established the molecular basis for this effect by showing that this polymorphism constrains both the propagation of distinct human PrP^Sc^ conformers and the occurrence of associated patterns of neuropathology [25,26,28,49,122,171]. Biophysical measurements suggest that this powerful effect of residue 129 on prion strain selection is likely to be mediated via its effect on the conformation of PrP^Sc^ or its precursors or on the kinetics of their formation, as it has no measurable effect on the folding, dynamics or stability of PrP^C^[5,174]. Heterozygosity at codon 129 is thought to confer resistance to prion disease by inhibiting homologous protein-protein interactions essential for efficient prion replication [80,81,171,172] while the presence of methionine or valine at residue 129 controls the propagation of distinct human prion strains via conformational selection [2,5,14,49]. To date, the repertoire of PrP^Sc^ isoforms that can be stably propagated by human PrP with 129 methionine or 129 valine remains unknown.

## Transgenic modelling of inherited prion disease

How pathogenic mutations in *PRNP* cause prion disease has yet to be resolved; however, in most cases the mutation is thought to lead to an increased tendency of PrP^C^ to form PrP^Sc^. However, there is now considerable evidence that different mutations may have different structural consequences in the expressed protein, including acting to destabilize the native PrP^C^ fold, to increase aggregation propensity, to alter cellular trafficking, or to stabilize alternative protein (PrP^Sc^) structures [175–180]. While a wealth of data from acquired or sporadic CJD indicates that residue 129 polymorphism critically dictates thermodynamic preferences for PrP^Sc^[2,5,49,52,90], the full spectrum of effects that different pathogenic *PRNP* mutations have remains unclear. However, molecular strain typing has provided important insights into the phenotypic heterogeneity seen in inherited human prion diseases. In agreement with existing evidence that human prion strain diversity may be generated through variance in PrP^Sc^ conformation and glycosylation, cases of inherited prion disease caused by point mutations have glycoform ratios of PrP^Sc^ fragments distinct from those seen in both classical CJD [103,177,181–183] and vCJD [177]. Individuals with the same *PRNP* mutation can also propagate PrP^Sc^ with distinct fragment sizes [103,177,184]. However, the detection of PrP^Sc^ in the molecular mass range of *c*. 21–30 kDa is by no means a consistent feature and some *PRNP* mutations, in particular those in which amyloid plaques are a prominent feature, show smaller protease resistant fragments of *ca*. 7–15 kDa [72,103,177,181,184,185] while other *PRNP* mutations show a consistent absence of detectable PrP^Sc^[2,72]. Collectively, these data indicate that pathogenic *PRNP* mutations have diverse and direct effects on dictating the preferred structure or assembly state of mutant PrP^Sc^ isoforms resulting in physicochemical properties that are very different from the PrP^Sc^ types propagated in sporadic and acquired forms of human prion disease [74,177,186]. Notably, variable propagation of PrP^Sc^ generated from wild-type PrP^C^ may also contribute to phenotypic variability in inherited prion disease [74,187–189]. Co-propagation of distinct PrP^Sc^ types combined with differences in their neuropathological targeting, abundance and potential neurotoxicity, provides a general molecular mechanism for generating phenotypic heterogeneity in patients with the same *PRNP* mutation.

Attempts to transmit inherited prion diseases to nonhuman primates [57] and wild-type mice [190–192] have been inconclusive in answering whether all inherited prion diseases are experimentally transmissible as nonhuman hosts may not be susceptible. While some inherited prion diseases may indeed not be transmissible, and may represent prion proteinopathies [193–195], many pathogenic mutations have yet to be tested in transgenic mice expressing the homotypic human mutant protein. This may be critical as only the human mutant protein may be conformationally susceptible to the mutant prion strain involved [5,186]. Much of the transgenic modelling of inherited prion disease has however focused on superimposing human PrP mutations onto rodent PrP^C^ in order to establish whether infectious prions can be generated *de novo*. To date, spontaneous neurological dysfunction has been reported in multiple transgenic models expressing mutated rodent PrP. These include mice expressing mouse PrP P101L [196], mouse PrP with octapeptide repeat insertions [194,197–199], truncated mouse PrP [131,200], mouse PrP with D177N and M128V substitutions [201], mouse PrP with L108M, V111M and D177N substitutions [202] and transgenic mice expressing a counterpart of the human A117V mutation or experimental mutations that favour the generation of a transmembrane form of PrP [193,203].

Of the various *PRNP* mutations studied, the proline to leucine substitution at codon 102 (P102L) of human PrP has been extensively investigated in different laboratories. However, these data have been difficult to interpret. Tg(GSSPrP)174 mice expressing high levels of mouse PrP 101L spontaneously develop neurological dysfunction at 166 days of age [196], however, PrP^Sc^ levels are low or undetectable, and brain extracts from affected mice do not transmit CNS degeneration to wild-type mice, but transmission to hamsters and Tg(GSSPrP)196 mice, expressing lower levels of the same mutant transgene product, was reported [204,205]. However, these Tg(GSSPrP)196 mice have subsequently been reported to develop spontaneous disease at advanced age [110,112] and it therefore remains inconclusive whether transmissible prions were generated in these transgenic mice or that the illness observed on secondary passage simply represents acceleration of a spontaneous neurodegenerative disease that is already poised to occur [65,112]. Importantly, in this regard, transgenic mice expressing endogenous levels of mouse PrP 101L (generated by the gene knock-in approach) do not develop spontaneous neurodegeneration [165,192] while mice over-expressing wild-type PrP^C^ have been found to develop spontaneous neurological dysfunction without generating infectious prions [116,206–208].

Collectively, the existing data have yet to conclusively establish whether authentic high titre infectious prions have been generated *de novo* in mice expressing mouse PrP containing only human pathogenic mutations. In this regard, the critical step of showing transmission to wild-type mice on primary passage remains. An extremely important consideration in such studies is whether superimposition of pathogenic human PrP mutations into rodent PrP will have the same structural consequences. Indeed, there are now examples of inherited prion disease where the amino acid change thought to be pathogenic is found as a normal variant in other mammalian species [209–211] and critically there is now direct experimental evidence indicating that a single analogous amino acid change in human or mouse PrP has extremely different structural consequences for the expressed protein. The introduction of a tryptophan residue at amino acid position 175 in place of the native phenylalanine has been successfully used as an optical probe for studying the folding dynamics of recombinant mouse PrP with no measurable effect on the stability of the protein [212]. However, in complete contrast, introduction of the same mutation into human recombinant PrP renders the protein unable to fold into the native conformation [213]. These findings clearly raise doubt about modelling human pathogenic PrP mutations on nonhomologous PrP sequences from other species. The possibility of propagating novel prion strains that do not recapitulate the molecular and neuropathological phenotype of the original human disease appears probable and for this reason it seems clear that future transgenic models of inherited prion disease should focus on expressing mutated human PrP [186,214].

Of course, studies of the effects of experimental mutations on mouse PrP should also continue. A recent report of *de novo* generation of prion disease in such models involved the introduction of 2-point mutations into mouse PrP (170N and 174T) that are found as normal variants in the rigid loop of elk PrP [215]. Transgenic mice mPrP(170N,174T), moderately over-expressing these mutations spontaneously develop spongiform encephalopathy and PrP plaque deposition in the brain [215]. Repeated subpassages in Tg20 mice showed transmission of disease, after adaptation, to wild-type mice by the fourth passage, and propagation of protease resistant PrP^Sc^[215]. Recently, Lindquist and colleagues have reported that mice expressing mouse PrP with L108M, V111M and D177N substitutions generated by knock-in technology spontaneously produce transmissible prions [202].

## Difficulties associated with modelling human prion disease in human PrP expressing transgenic mice

Whether the full diversity of neuropathological phenotypes seen in human prion disease can be faithfully recapitulated by transgenic modelling remains an open question. In this regard the issue of prion strain selection or mutation will be a major factor. As recently hypothesized [5] prion strains may not exist as previously thought as molecular clones with a single PrP^Sc^ type (where strain mutation in a different host would involve generation of a distinct PrP^Sc^ type) but may consist of an ensemble of molecular species (containing a dominant PrP^Sc^ type that is preferentially propagated by its usual host) from which a less populous subspecies may be selected by an alternative host, resulting in a strain shift or mutation. Different cellular populations and tissues within a single host would provide different environments for strain selection as recently demonstrated *in vitro*[216]. In addition, the known ability of genetic background to influence prion strain selection [28,50,51,117–119] means that it may be extremely difficult to isolate the full complement of human prion strains in transgenic mice having a single genetic background.

## Future perspectives

To date, the conformational repertoire of pathological isoforms of wild-type human PrP and the various forms of mutant human PrP has not been fully defined. Biochemical investigation of PrP^Sc^ isoforms in patients allied with detailed clinical and neuropathological analysis will continue to inform on the diversity of phenotypes seen in human prion disease. As it has now become clear that prion strain type, host genetic makeup and other factors (e.g. route of transmission) may significantly influence prion disease phenotype it is expected that the actual number of distinct human prion strains may be far less than the number of identified phenotypes. Continued transgenic modelling will therefore be crucial to establishing how many human prion strains exist, and what the defining molecular features of PrP^Sc^ are for each strain. This information allied with comprehensive transgenic modelling of human BSE infection and other relevant, potentially zoonotic, prion strains will inform on how many human prion strains may have an animal origin. Understanding the risks that existing and emerging animal prion diseases pose will have direct translation to protecting public health.

Development of an accurate classification for human prion disease will have major implications for epidemiological research into the causes of sporadic CJD, whose aetiology remains obscure. While spontaneous conversion of PrP^C^ to PrP^Sc^ as a rare stochastic event, or somatic mutation of the PrP gene, resulting in expression of a pathogenic PrP mutant are plausible explanations for sporadic CJD [2,74,217,218], other causes for at least some cases, include environmental exposure to human prions [219–221] or exposure to animal prions. In this regard, the number of prion strains causing sheep scrapie has yet to be established [43,45,46] and epidemiological data cannot exclude this as a cause of a proportion of cases. As future research begins to provide a more precise understanding of the origins of human prion disease, this will facilitate re-analysis of epidemiological data, to reveal important risk factors that might have been obscured by analysing sporadic CJD as a single entity.

While much remains to be done in addressing fundamental questions about human prion strains, transmission barriers, subclinical carrier states and the role of PrP polymorphisms and mutations in the aetiology of these diseases and the production of prion strains, a key development in the future will be application of these highly characterized models to evaluate candidate therapeutic drugs and antibodies [158,159,222]. In addition, it is now becoming increasingly clear that genetic loci other than *PRNP* may play a significant role in prion pathogenesis and strain selection [117,118,223,224]. Long-term genomic studies in both mouse and human have recently identified a number of genes affecting prion disease incubation period or susceptibility [50,51]. The characterization of such genes in new transgenic models is expected to cast significant light on pathogenic mechanisms, including prion co-factors, and may identify new therapeutic strategies.
